# On the Action of Tincture of Perchloride of Iron in the Cure of Renal and Urinary Affections

**Published:** 1865-06

**Authors:** Arthur Hill Hassall

**Affiliations:** London


### ON THE ACTION OF TINCTURE OF PERCHLORIDE OF IRON IN THE CURE OF RENAL AND URINARY AFFECTIONS.

BY ARTHUR HILL HASSALL, M. D., LONDON.

There are few remedies more frequently prescribed in the treatment of renal and urinary affections than is the tincture of perchloride of iron, formerly called muriated tincture of iron. The value of that remedy in a variety of such cases is undoubted, as in the albuminuria of Bright's disease, in hemorrhage from the kidney, bladder, or urethra, in spasmodic stricture, etc.

The tincture of the perchloride of iron consists of two atoms of iron in combination with three of chlorine, dissolved in water to which rectified spirit has been added; and it possesses the properties of an astringent, tonic, and styptic, coagulating blood or albumen with which it is brought into contact, and constringing the vessels and tissues to which it is applied.

Now, this astringent property is just that which a remedy ought to possess to be useful in the cases above referred to, and by it, it is usually supposed, it exerts its beneficial action; and certainly nothing would appear to be more plausible and more natural than this explanation.

Having, after the administration of this remedy, repeatedly tested the urine for the purpose of detecting in it the presence of iron, and having failed to discover the faintest trace of the metal, I was led to doubt the correctness of this view, and was induced to institute some experiments, in order to put the matter to the proof.

To a patient, T. L——, laboring under an habitual urinary discharge, I administered for the period of more than a week a drachm of the tincture thrice daily; but, although I tested the urine on several occasions, in no instance could I detect the smallest trace of iron, notwithstanding that a pint of the urine was evaporated to a small bulk before being examined.

To a patient now in the Royal Free Hospital, Charles S——, who is suffering from an extravasation of blood, three drachms of the tincture were administered on two consecutive days. The whole of the urine passed in the twenty-four hours of each day was collected, a pint of each sample evaporated to a small bulk, and tested as before, but with a similar negative result.

Lastly, I myself took in the course of a day three drachms of the tincture; the urine passed in the twenty-four hours being collected and analyzed, not only on the day on which the medicine was taken, but on the preceding and succeeding days. Still no iron was found.

I could enumerate several other instances in which iron had been taken and the urine analyzed without even traces of the metal being subsequently discovered. The examples, however, I have quoted are sufficient to show that the tincture of perchloride of iron does not produce its beneficial effects, as generally supposed, in restraining the amount of albumen or of blood discharged from the kidney or other portion of the genito-urinary mucous track by coming in contact with the seat of the lesion and by its action as an astringent.

How, then, does this remedy act? That much of the iron contained in the sesquichloride does not find its way into the circulation at all, but escapes from the system with the undigested portions of the food, is certain; the black discoloration of the fæces under the use of this tincture, and indeed, I believe, under all the preparations of iron, is well known, the color being due to a combination of the iron with a portion of the sulphur of the food—sulphuret of iron being thus formed. It might therefore be very plausibly presumed that while the greater part of the iron is thus thrown off by the bowels without having been absorbed at all, the hydrochloric acid, being set free, enters the circulation, is eliminated by the kidneys, and so comes in contact with the seat of lesion; and that it is to the acid, and not to the iron, that the benefit is to be attributed. But if this view be correct, it is capable of being substantiated by experiment; and with this object I administered to two persons drachm doses, repeated thrice daily, of the perchloride; the urine of the twenty-four hours being collected and analyzed before, during, and after the administration of the ferruginous preparation. * * * * * *

The experiments showed, 1st, that there was no increase in the acidity of the urine consequent upon taking the remedy; 2d, that there was no increase of chlorine, and that therefore the hydrochloric acid of the perchloride was not eliminated by the kidneys either in the free or combined state; thus proving that the second view mentioned of the action of the remedy is also entirely unfounded.

These results are not a little remarkable; and we have, therefore, still to inquire, in what way does this medicine act in the cure of disease? Its effects are too rapid to allow it to be supposed that its operation is due to its influence in improving the condition of the blood by its action on the red corpuscles. We appear, therefore, driven to the conclusion that the perchloride of iron acts by its stimulating influence on the nervous system.

These observations are interesting, not alone as concerns this one preparation of iron; they also probably apply more or less to most of the other medicinal preparations of that metal, since it is at least certain that by far the greater part of the iron contained in them is not absorbed, but escapes from the system by the bowels like that of the perchloride.

The particulars herein recorded are suggestive of further experiments calculated to throw additional light upon the subject, and which hereafter I may have the opportunity of instituting.

**Wimpole street, 1865.**

---

*To the Editor of The Lancet:*

Sir:—I was much interested in Dr. Hassall's valuable and suggestive paper on this subject, published in *The Lancet*, in which he mentions that the efficacy of perchloride of iron depends on its stimulating influence on the nervous system, especially referring to its use in the cure of renal and urinary affections.

In former notes I have spoken from practical experience of its value in the treatment of erysipelas, venturing to differ from so high an authority as Dr. Hughes Bennett, who says it is useless in this disease.

I believe that the tonic, or rather the roborant and *stimulating* influence of the remedy is the rational way of accounting for its good effect in erysipelas, in the treatment of which it has now been used for several years; and we have here an explanation of its efficacy in delirium tremens, in which it has lately been employed with remarkable success.

This medicine is also much used in the treatment of phthisis, diphtheria, cynanche, etc., and I think it is generally admitted that the chlorine which it contains, as well as the iron, acts with peculiar benefit in these diseases.

I hope Dr. Hassall may have an opportunity of instituting further experiments on this subject, which is far from exhausted.*

I am Sir, your obedient servant,

JOHN ROSE, M. D., Surgeon R. N.

Kidderminster, January 2, 1865. [*Lancet.*

* It is desirable above all, if it has positive influence over any of these diseases—erysipelas, delirium tremens, phthisis, diphtheria or cynanche, that this be more certainly determined. It is a common remedy, but its effects are not gene.ally very obvious.—[ED. BUFFALO MED. JOUR.

